# A High-Resolution Mass Spectrometry-Based Quantitative Metabolomic Workflow Highlights Defects in 5-Fluorouracil Metabolism in Cancer Cells with Acquired Chemoresistance

**DOI:** 10.3390/biology9050096

**Published:** 2020-05-06

**Authors:** Sanjay Shahi, Ching-Seng Ang, Suresh Mathivanan

**Affiliations:** 1 Department of Biochemistry and Genetics, La Trobe Institute for Molecular Science, La Trobe University, Melbourne, VIC 3086, Australia; 18604885@students.latrobe.edu.au; 2 The Bio21 Molecular Science and Biotechnology Institute, University of Melbourne, Parkville, VIC 3010, Australia

**Keywords:** 5-Fluorouracil, metabolites, precursor isotopes, single ion monitoring, high-resolution mass spectrometry, nucleotide analogue, chemoresistance, colorectal cancer

## Abstract

Currently, 5-fluorouracil (5-FU)-based combination chemotherapy is the mainstay in the treatment of metastatic colorectal cancer (CRC), which benefits approximately 50% of the patients. However, these tumors inevitably acquire chemoresistance resulting in treatment failure. The molecular mechanisms driving acquired chemotherapeutic drug resistance in CRC is fundamental for the development of novel strategies for circumventing resistance. However, the specific phenomenon that drives the cancer cells to acquire resistance is poorly understood. Understanding the molecular mechanisms that regulate chemoresistance will uncover new avenues for the treatment of CRC. Among the various mechanisms of acquired chemoresistance, defects in the drug metabolism pathways could play a major role. In the case of 5-FU, it gets converted into various active metabolites, which, directly or indirectly, interferes with the replication and transcription of dividing cells causing DNA and RNA damage. In this project, we developed a high-resolution mass spectrometry-based method to effectively extract and quantify levels of the 5-FU metabolites in cell lysates and media of parental and 5-FU resistant LIM1215 CRC cells. The analysis highlighted that the levels of 5-FU metabolites are significantly reduced in 5-FU resistant cells. Specifically, the level of the nucleotide fluorodeoxyuridine monophosphate (FdUMP) is reduced with treatment of 5-FU clarifying the compromised 5-FU metabolism in resistant cells. Corroborating the metabolomic analysis, treatment of the resistant cells with FdUMP, an active metabolite of 5-FU, resulted in effective killing of the resistant cells. Overall, in this study, an effective protocol was developed for comparative quantitation of polar metabolites and nucleotide analogues from the adherent cells efficiently. Furthermore, the utility of FdUMP as an alternative for CRC therapy is highlighted.

## 1. Introduction

Colorectal cancer (CRC) is the third most common cancer and has the fourth highest mortality rate worldwide [1,2,3] The incidence of CRC is highest in Australia/New Zealand and Western Europe. Surgery is the main treatment option for early stage CRC patients [4]. If metastasis has occurred (stage IV), chemotherapy is the first line of treatment to prolong and maintain the quality of life of the patients [5,6]. The main chemotherapeutic drugs for CRC treatment are 5-fluorouracil (5-FU), irinotecan and oxaliplatin [7]. 5-FU has been used for the treatment of various cancers for more than 45 years and is the most commonly used standard first-line chemotherapeutic drug for CRC treatment [8,9]. In the metastatic setting, the response rate to 5-FU when used alone is approximately 15% [7,9]. However, most of these patients eventually acquire resistance to 5-FU, resulting in treatment failure and patient death. While a combination of 5-FU with other anti-cancer drugs (irinotecan and oxaliplatin) has resulted in higher response rates (40–50%), it is crucial to understand the molecular mechanisms regulating chemoresistance to 5-FU in order to predict or overcome drug resistance. Unravelling the mechanisms of 5-FU resistance could result in the stratification of patients likely to respond to this agent. Furthermore, it may aid in the identification and development of therapeutic strategies aimed at preventing or overcoming resistance.

5-FU metabolism involves the sequential conversion of 5-FU into fluorouridine (FURD), fluorodeoxyuridine (FdURD), fluorodeoxyuridine monophosphate (FdUMP), fluorouridine triphosphate (FUTP) and fluorodeoxyuridine triphosphate (FdUTP) inside the cells. FUTP and FdUTP are incorporated into DNA and RNA causing disruption in transcription and translation, and hence, leading to cell death. The active metabolite FdUMP is involved in the inhibition of the deoxythymidine triphosphate (dTTP) synthesis leading to the accumulation of deoxyuridine monophosphate (dUMP), thereby causing a disruption of replication by direct incorporation of dUMP instead of the dTTP [7]. Despite the anti-cancer properties of 5-FU, chemoresistance creates significant obstacles in treating cancer patients. Drug resistance mechanisms in general are thought to be regulated by molecules that are involved in evading apoptosis, decreased uptake of drugs, increased efflux, inactivation of drugs, overcoming oxidative stress, increased damage repair and altering cell cycle checkpoints [10,11]. In case of 5-FU, dysregulation of uridine monophosphate synthetase (UMPS), an essential enzyme for uridine biosynthesis and conversion of 5-FU, has been linked with reduced expression of the Bcl2 like ovarian killer (BOK) protein in CRC cells, which are resistant to 5-FU treatment [12]. Similarly, increased expression of the enzyme thymidylate synthase [13] and dihydropyrimidine dehydrogenase [14] have also been implicated in drug resistance. However, the knowledge on the role of the 5-FU metabolism pathway and all its metabolites in acquired chemoresistance has been limited. This scarce knowledge can be attributed to the lack of robust metabolic workflows to quantify 5-FU metabolites without standards in cells and conditioned media.

There are multiple methods for the detection and the analysis of cellular nucleotides. Many approaches that involve the detection and analysis of such compounds from the mixture of cellular components involve liquid chromatography coupled with mass spectrometry (MS). Traditionally, the MS component involves a triple quadrupole for its high sensitivity and specificity, yielding lower detection, thus, lower quantitation limits [15]. However, selectivity may sometimes be overestimated due to the inherent low resolution and mass accuracy of these instruments, thus, resulting in false positives [16]. As the cells are a highly complex medium consisting of proteins, nucleic acids and metabolites, complications arise when attempting to identify these components using MS among a huge magnitude of different components of similar molecular weight, charge states and physiochemical properties. Hence, sample processing and preparation potentially complicates the identification. Furthermore, the matrix effect, originating from pre-treatments, processing and contaminations, make the identification and quantification extremely challenging [17,18]. With advances in MS, high-resolution MS (HRMS) combined with ultra high performance liquid chromatography (UHPLC) have been gaining popularity, and hence, have been widely used for the identification of compounds from complex samples [19,20]. Using hyphenating acquisition methods such as Targeted Single Ion Monitoring (tSIM) coupled with data dependent MS2 (ddMS2) [21,22] can further help us overcome some of the quantitation issues in highly complex samples. Additionally, past studies performed for the quantification of 5-FU and metabolites looked at single sample and were dependent on the availability of standards [23]; hence, attempts to provide an efficient way to detect and quantify small molecules from complex samples when in the absence of standards are limited. In addition, a normalized comparison of 5-FU and metabolites among two individual group of adherent cells (resistant and parental) also need to be addressed.

In this study, we developed a high-resolution mass spectrometry (MS)-based workflow for the effective extraction, detection and quantification of the polar metabolites to study the role of 5-FU metabolism in chemoresistance. The levels of various metabolites could be quantified in cell lysates and conditioned media. The analysis highlighted the elevated levels of the nucleotide dUMP in 5-FU resistant CRC cells, thereby suggesting a disruption in 5-FU metabolism. The treatment of the resistant cells with FdUMP induced apoptosis in the 5-FU resistant CRC cells. Taken together, for the first time, we report an effective protocol to quantify polar metabolites and nucleotide analogues and highlight the utility of FdUMP as an alternative in sensitizing 5-FU resistant CRC cells.

## 2. Materials and Methods

### 2.1. Cell Culture, Generation of 5-FU Resistant Cells

Parental LIM1215 CRC cells were obtained from Prof. John Mariadason, Ludwig Institute of Cancer Research, Melbourne and were maintained in RPMI 1640 (GIBCO, Life Technologies, Waltham, MA, USA) medium supplemented with 10% (v/v) FCS (fetal calf serum) (GIBCO, Life Technologies) and 100 Units/mL penicillin-streptomycin antibiotics (GIBCO, Life Technologies) in a 37 °C incubator and under 10% CO_2_. 5-FU resistant LIM1215 cells were generated by continuous culture of parental CRC cells in increasing concentrations of 5-FU. The cells were seeded and cultured up to 70% confluency, and then, the media was replaced with media containing 1 µM 5-FU and cultured until 100% confluent. The cells were then trypsinized and reseeded with an increased concentration of 5-FU (5 µM). The cells were cultured until 100% confluency was reached, with a fresh treatment media with drugs supplied every 3 days. The cells were then trypsinized and the process was repeated with 10, 25 and, finally, 50 µM 5-FU, respectively. For the metabolomics analysis, cells were cultured in 150 mm^2^ cell culture dishes (BD Falcon^TM^) to 80–85% confluency and the cell lines were supplemented with 14 mL culture media with and without 50 µM 5-FU and further incubated at 37 °C for 72 h with 5% CO_2_.

### 2.2. FACS Cell Death Assay

An equal number of cells were seeded in 24-well plates and incubated overnight at 37 °C with 5% CO_2_. The plates were then treated with 50 μM 5-FU and incubated for 72 h. Following treatment, cells were scrapped off gently using a plastic scrapper and the cell suspension was collected in 96-well plates. The plates were centrifuged at 1500× *g* at 4 °C for 5 min using an Allegra^®^ X-15R centrifuge (Beckman Coulter, Brea, CA, USA) to pellet the cells. Supernatants were discarded, and cells were stained overnight at 4 °C with an addition of 2.5 μg/mL propidium iodide (PI) buffer (Life Technologies). Flow cytometry was performed using FACSCanto (Beckton Dickinson, Franklin Lake, NJ, USA) and for each sample 30,000 events were collected and cell death (% PI positive cells) was analyzed by using the FlowJo^®^ program (FlowJo, LLC, Beckton Dickinson). For FdUMP treatment, the same protocol was used, substituting 5-FU with FdUMP.

### 2.3. Extraction of 5-FU and Its Metabolites from Media

Conditioned media were collected, and dead cells and debris were removed by centrifugation at 1500× *g* for 5 min. Supernatants were mixed with 99.9% (v/v) ice-cold molecular grade acetone at a ratio of 1:5 in a 50 mL centrifuge tubes and incubated overnight at −20 °C for protein precipitation. Samples were then subjected to initial centrifugation at 1700× *g* for 5 min (Haraeus Megafuge 1.0) and the supernatants were collected and further centrifuged at 12,000× *g* for 10 min at 4 °C to precipitate out the proteins (SW28 rotor in Beckman Coulter Optima^TM^ L-100XP Ultra centrifuge). For this purpose, thin-wall polypropylene centrifuge tubes (Beckman Coulter) were used. Supernatants were concentrated using a Speed vac concentrator (SAVANT SPD 131 DDA, Thermo Scientific, Waltham, MA, USA) to first remove the more volatile acetone. The protein depleted samples were then freeze dried (Martin Christ Beta 2–8 LD Plus) and stored at −80 °C until further processing and analysis.

### 2.4. Extraction of 5-FU and Its Metabolites from Cells

LIM1215 cells are adherent and grown as a monolayer in the culture dishes. Following collection of media from treated and untreated cell culture dishes, the cells were washed thrice with 1× PBS (137 mM NaCl, 10 mM phosphate, 2.7 mM KCl, pH 7.4) for removal of metabolites and 5-FU present outside cells. Cells were collected by lifting off with trypsin and resuspending in complete media to neutralize trypsin followed by centrifugation at 1500× *g* for 5 min. Pellets of cells obtained were resuspended in the fresh media and subjected to Trypan blue assay by staining cells with 0.4% (v/v) Trypan blue (Santa Cruz) and counting in a hemocytometer.

Cells (5.25 × 10^7^) were harvested and centrifuged at 1500× *g* for 5 min. The cell pellet was quickly washed with 5 mL ice-cold PBS, then centrifuged again to pellet them down. Metabolites and 5-FU were extracted by using mechanical cell lysis technique. As 5-FU and its metabolites are polar, the cells were resuspended in 1 mL 100% (v/v) methanol and subjected to three repeated cycles of snap freezing with liquid nitrogen for 10 min, then thawed at room temperature and occasional vortexed (Vortex V-1 Plus, Scientifix), followed by sonicating for 10 min in a waterbath sonicator (Bransonic 12, Branson). The samples were then incubated in a −80 °C freezer for 2 h and subjected to centrifugation at 12,000× *g* for 10 min at 4 °C (Velocity 15µR Microcentrifuge, Dynamica). The supernatants were collected and concentrated using a Speed vac concentrator (SAVANT SPD 131 DDA, Thermo Scientific) for the complete removal of methanol followed by freeze drying (Martin Christ Beta 2–8 LD Plus) and stored at −80 °C.

### 2.5. Preparation of Media and Cell Extract Samples for Mass Spectrometry Coupled with Reverse Phase UHPLC

The freeze-dried media and cell extract samples were dissolved in equal volume, 200 µL and 400 µL, respectively, of double distilled deionized water. To ensure complete solubilization, samples were subjected to vortexing for 5 min using the Vortex V-1 Plus (Scientifix) and sonication for 10 min (Bransonic 12, Branson) thrice, each followed by centrifugation at 18,000× *g* for 30 min using a Velocity 15µR Microcentrifuge (Dynamica). Supernatants were transferred to mass spec vials for analysis.

### 2.6. Ultra High Performance Liquid Chromatography Coupled with High-Resolution Mass Spectrometry

Pentafluorophenyl F5 phase chromatography was used for the separation of the polar compounds from mixture of cellular and biological samples [24,25]. 10 µL of standards, 10 µL of media and 30 µL of lysate samples were injected through a Vanquish UHPLC system (Thermo Fisher) coupled to an Orbitrap Q Exactive HF Hybrid Quadrupole-Orbitrap MS (Thermo Fisher). For chromatographic separation, a pentafluorophenyl F5 column (Kinetex F5 Coreshell 1.7 µm, 100 × 2.1 mm, Phenomenex) was used. Buffer A was 10 mM ammonium formate (pH4) in water and B was methanol. The flow rate was 0.2 mL/min, flow gradient was (i) 0–3 min, 0–5%B, (ii) 3–4 min, 5–100%B, (iii) 5–5.1 min, 100–0%B and equilibrated at 0%B for 3 min before the next injection.

The targeted single ion monitoring, data dependent MSMS (tSIM-ddMS2) methodology was applied with the below settings (Table 1). An inclusion list for eight of the targeted compounds was used to trigger fragmentation on the precursor mass (Table 2). MS was performed in negative ion mode and electron spray ionization (ESI) introduced the samples to the first quadrupole as [M-H]^-^ ions of the compounds. The precursor ions of the specific mass ranges matching to the m/z of target compounds were isolated and passed to second quadrupole where they were subjected to high energy collision-induced dissociation (HCD). HCD dissociated these precursors to form fragment ions of a specific m/z ratio, giving rise to specific MSMS (MS2) fragmentation spectrum, characteristic for specific products compounds. The raw data were obtained and analyzed using different standalone tools.

### 2.7. Preparation of 5-FU and FdUMP Standards for Generation of Calibration Curve to Determine LOD and LOQ

Stock 5-FU and FdUMP standards were prepared in 100% (v/v) methanol and 1× PBS, respectively, whereas working solutions were prepared in double distilled deionized water. These standards were serially diluted in water and 10 µL of the solutions were injected for analysis. To mimic the biological sample condition and factor for matrix suppression, standards were serially diluted in concentrations from 100 ng/µL to 0.195 ng/µL in processed conditioned media and analyzed. We first optimized the separation condition to obtain the sharpest peak shape and resolution of the eluted peak from the F5 column followed by the collision energy setting on the MS in water followed by the growth media. The precursor ion peak (m/z values) from the MS1 spectrum, including the retention time and the fragment ion peaks (m/z values) of these standards, were recorded. These precursor and fragment ion peaks were then used as a reference in the test samples to detect and quantify 5-FU and FdUMP. The results were processed using the Skyline v19.1 software (see below) and the processed results used to generate the calibration curve using GraphPad Prism 8 software. From the calibration curve of FdUMP, one specific peak area obtained from 0.780 ng/µL concentration standard (within the linear range), was selected for the relative quantification of all compounds. For normalization purposes, the FdUMP spiked QC sample was analyzed once every set of biological replicates. Blanks runs were carried out extensively to ensure no carryover of analytes.

### 2.8. Identifying and Quantifying 5-FU and its Associated Metabolites

#### 2.8.1. Selection of Precursor Mass for tSIM-ddMS2 and Detection of 5-FU and Its Associated Metabolites in Samples Using Accurate Precursor Mass

For 5-FU and FdUMP, the measured precursor m/z, fragment ion m/z and retention time information was acquired first. For compounds without standards available (TMP, dUMP, FURD, FdURD, FUTP and FdUTP), the target precursor m/z was calculated for each using the isotope simulation in Xcalibur Qual browser first as follows:

(a) Molecular formula of the compounds were first entered in the isotope simulation section with the deduction of one Hydrogen (H) from the formula and the software gave the m/z as well as the isotope pattern as shown in Supplementary Figure S1 for FURD and Supplementary Figure S2 for FdUMP.
(b) An m/z [M-H]^-^ targeted inclusion list (Table 2) of all the eight precursor masses was then used as a target for the tSIM-ddMS2 experiment.
(c) For data analysis, specific mass range filter in Qual browser was used to view the elution of the compounds that had similar m/z of target precursor at a 5 ppm mass error.
(d) Within this broad range of mass filter, an additional mass filter was applied, which was accurate up to three decimal points, as shown in Table 2 for each compound. This helped in the reduction of nonspecific peaks and limited the search to a definite range around the target [26] to get the clear peak of the target precursor at specific retention time, as shown in Supplementary Figure S1A.

#### 2.8.2. Selection of Fragment Ions (Transitions) for Identification and Quantification of 5-FU and Its Associated Metabolites

For 5-FU and FdUMP, standard samples were available; hence, the transition ions were obtained by performing the MS analysis of standard samples and the fragment ions were analyzed. The fragment ions were selected from the experimentally observed spectrum and compared with previous literature. For compounds without standards, fragment ions were selected based on the published literature discussing the fragmentation of these specific compounds from spectral databases such as mzCloud [27,28,29], NIST [30,31] and the Human metabolome database (HMDB Version 4.0 [23,32,33]) or through theoretical prediction using different tools. For prediction of theoretical fragment ions, we utilized a competitive fragmentation modelling (CFM) and machine learning, CFM-ID approach [34,35].

To perform the prediction;

(a) The ‘canonical SMILES’ formula were first obtained from PubChem [36].
(b) To generate theoretical spectra, the canonical smiles formula was subjected in the compound structure section within the spectra prediction utility of the CFM-ID 3.0 online tool.
(c) The search was processed with Spectra type ‘ESI’ with negative ion mode and [M-H]- adduct type from the drop-down menus.

The canonical SMILES formula identified fragmentation peaks/compounds (theoretically generated using CFM-ID tool and as found in spectral database as HMDB) are shown in Supplementary Figure S3. These fragment ions were then compared with the previously published literature data, where available [23,37].

#### 2.8.3. Detection of 5-FU and Associated Metabolites Using Accurate Precursor and Fragment Masses

Following detection of potential precursors from its accurate MS1 mass, the fragmentation spectrum of specific target precursor was visualized using the filter setting to MS2, obtained from specific target compound. The settings involved are:

(a) Selection of MS2 for specific m/z [M-H] shown in Table 2 at a retention time when the peak for precursor mass was observed
(b) The fragment ions from the MS2 spectrum were visualized in Xcalibur Qual browser software (Thermo Fisher) to ensure clean signal to noise, free from interfering signal.
(c) This way, in the same window, experimental data including the precursor along with fragment ion pattern obtained from it as well as the theoretically predicted isotope pattern using isotope simulation was visualized as shown in Supplementary Figure S1A.
(d) To validate that the precursors and fragments are detected correctly, the untreated test sample was run alongside and the absence of matching precursor as well as matching fragment ion patterns signified that the process was precise (Supplementary Figure S1B).

#### 2.8.4. Validation of the Correct Identification of the Compounds without Standards

We have applied an additional step to validate the unknown compounds. This is through isotope identification and MS fingerprint identification using the SIRIUS software [38]. SIRIUS 4.0.1 is a tool that can identify the molecular formula based on the measured precursor and the fragment ions from MS1 and MS2 independently, which were obtained from highly precise or sensitive equipment, such as Quadrupole-Time Of Flight (Q-TOF), Orbitrap and Fourier Transform-Ion Cyclotron Resonance (FT-ICR), which generated the high resolution and high accuracy data. The presence of the different isotopes in nature of certain compound based on the isotopic distribution of constituent elements gives rise to the slight variation in the m/z of the compounds from the calculated monoisotopic m/z of a precursor. Based on the frequency of the occurrence of such isotopes of constituent elements, the intensity pattern for the target precursor varies in the mass spectrum. SIRIUS identifies the compounds based on the presence of the different isotopes precursor; frequently available isotopes give higher intensity and it decreases with the availability of the isotopes, which is the basis of identification [39,40]. The fragmentation tree on the other hand represent the likelihood of the compound fragmenting into the predicted fragment ions, which can be explained as a part of the precursor ion. Scoring is based on different key properties: peak intensities, mass deviation among the prediction and the peak, chemical property of molecular formula, collision energy and the neutral loss occurring due to dissociation [41]. With the SIRIUS identification tool, the correct compound is usually identified in the results list obtained [42]. To perform SIRIUS analysis, the following steps were performed as instructed in the user manual [43];

(a) The MS1 spectrum was copied from the Qual Browser software by clicking in the spectrum and copying the data and creating an excel file in ‘.csv’ format.
(b) The precursor m/z data was obtained, and the file was then imported into SIRIUS 4.0.1 software following the steps mentioned in the user manual.
(c) The spectra file was dragged and dropped into the application window or alternatively imported using the import option. This gives the option to select the mass and intensity, where the default values are usually correct.
(d) MS level was selected (‘MS1′ for precursors or ‘MS2′ for product m/z list) and ’ok’ was clicked.
(e) The correct precursor molecular weight was selected from the dropdown option and the adduct was set to a negative ion format as ‘[M-H]’ and ‘ok’ was clicked to input the data.
(f) For analysis, we right clicked on the imported compound and compute was pressed. It is necessary to select the correct precursor ion mass in the dialogue box. For the compounds containing Fluorine, ‘F’ was added using the elements selection option, and the instrument was set to ‘Orbitrap.’
(g) A comparison was performed to check if the isotopic patterns matched the precursors isotopes pattern, and were then identified as the target compounds matching to PubChem database [40].
(h) The query was compared with the ‘all molecular formula’ option selected; however, SIRIUS generated a molecular formula with the elements that were selected in the compute dialogue box based on the ions m/z (precursor or fragment ions) and their mass, which might not be in the PubChem database. SIRIUS will consider all molecular formulas possible for the given specific precursor m/z, which will include the elements that are selected in the compute dialogue box [44].
(i) The isotope scores obtained for each target compound precursor identification step was then tabulated to form a prediction score table.
(j) To validate the fragment ions were fingerprint of the target precursors, the product mass spectrum data was copied from Skyline (Section 2.8.5) and as earlier, a .csv file was created.
(k) The file was then imported in SIRIUS to identify or predict the molecular formula. SIRIUS analyzed the fragments from MS2 and then identified the compound that these fragments might have been obtained from, and gave the score based on the identified peaks as explained in previous studies [38,41].

#### 2.8.5. Relative Quantification of 5-FU and the Associated Metabolites

The set of precursor mass and fragment ions or molecular fingerprints prepared for detection of compounds (Table 3) was prepared to be imported to Skyline v19.1 software. For the quantification of these compounds from test samples, a fingerprint composed of precursor and fragment ions for the individual compounds were used in Skyline software v19.1 [45] to identify compounds from test samples [46] as described in the instruction manual for small molecule analysis [47]. In Skyline software, the steps involved were:

(a) Inserting the transition within the insert menu.
(b) In the insert window, small molecules were selected instead of peptides, and then, the values (precursor name, precursor molecular formula, precursor adduct as M-H for all compounds) were added.
(c) Entry was checked for error using the option available within the software itself.
(d) The precursor was then inserted, which appeared in the target section of the Skyline window.
(e) Transition values representing the fragment ion m/z was then inserted for each precursor by following ‘right click’ in the precursor and then ‘add transition.’
(f) In this window, the transition m/z was typed in both monoisotopic and average m/z spaces with the adduct set to [M-] representing negative ions.
(g) To test the applicability of the approach, initial test samples data were obtained and analyzed following this procedure using Skyline and confirmed with XCalibur Qual Browser software (Thermo Fisher Scientific, version 3.1).
(h) Quantification was based on the peak area (total area, including precursor and products) obtained for each compound using skyline software from each sample. The peak area results were collected and stored in excel sheet for all the standards and one FdUMP standard (0.780 ng/µL) was chosen and all the compounds were normalized to the peak area of this standard.

As a second level of verification, the presence of the matching m/z ions in the test samples that were not observed in the untreated test samples at the specific retention time were checked as well. Furthermore, based on the properties of these compounds, we were able to detect these polar compounds in the earlier retention time only in the treated samples.

## 3. Results

### 3.1. Long Term Exposure of CRC Cells to Increasing Concentration of 5-FU Results in Acquired Resistance

**Figure 1:**
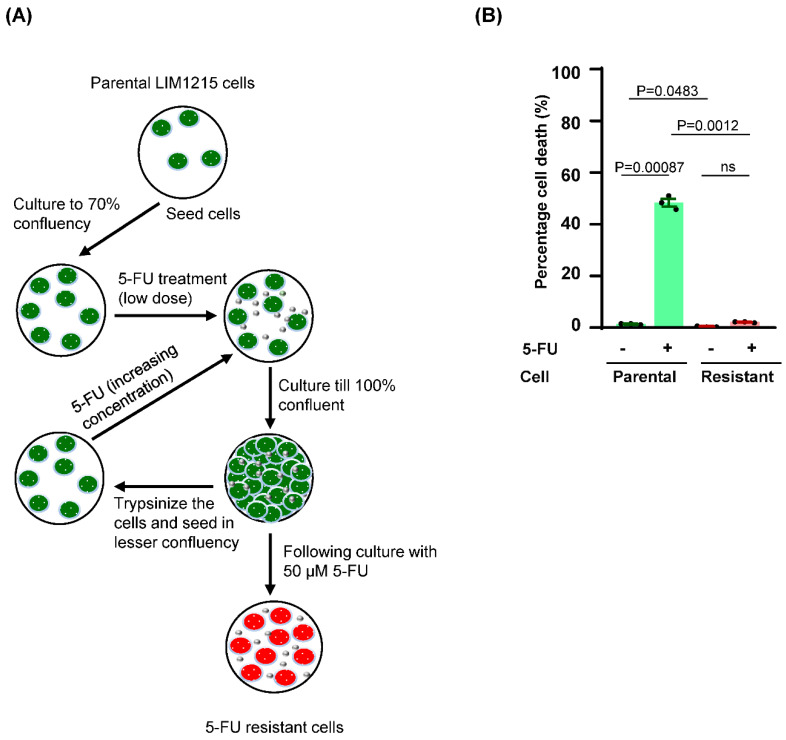
Colorectal cancer (CRC) cells gain resistance to 5-fluorouracil (5-FU) with long term exposure to 5-FU. (**A**) For developing 5-FU resistance in LIM1215 cells, parental cells were cultured continuously until they got fully confluent with increasing concentrations of 5-FU (1, 5, 10, 25 to 50 µm). (**B**) FACS apoptosis assay performed with LIM1215 parental and resistant cells following treatment with 50 µm 5-FU for 72 h showed that resistant cells are protected from 5-FU mediated cell death. ns; not significant. Data is presented as mean ± s.e.m.; significance determined by Student’s t-test (n = 3).

To elucidate the mechanism of chemoresistance via the inhibition of 5-FU metabolism pathway, a 5-FU resistant cell model was developed. LIM1215 CRC cells were cultured in the presence of increasing concentrations of 5-FU for extended period of time (Figure 1A). Follow up cell death analysis confirmed the induction of acquired resistance in the 5-FU resistant LIM1215 CRC cells (Figure 1B).

### 3.2. Calibration Using Known Standards

**Figure 2:**
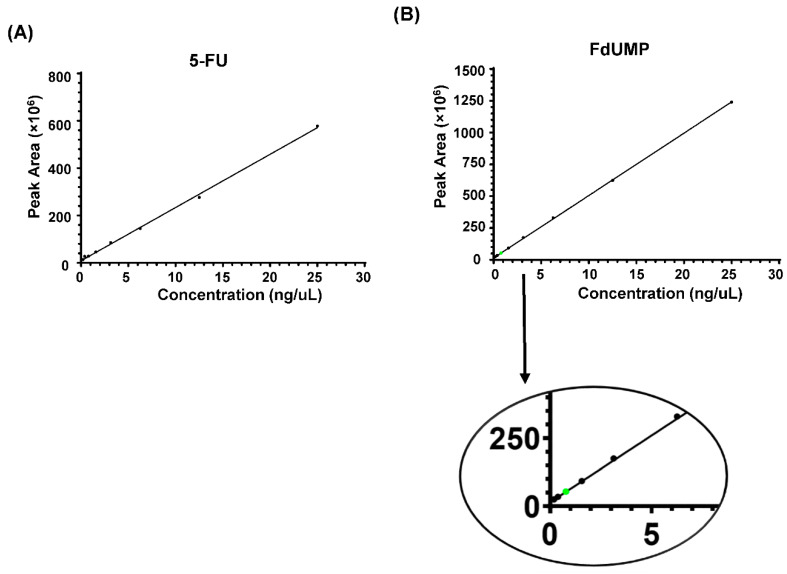
Generation of 5-FU and FdUMP calibration curve. Lowest limit of detection and quantification are two key factors for the accurate results. To determine the limits of detection and for the relative comparison of the results, the calibration curve was generated using the peak area obtained from serially diluted 5-FU (**A**) and FdUMP (**B**) spiked into conditioned media from the parental cell culture. The green dot from the curve represents the peak area of the 0.780 ng/µL concentration of FdUMP, which was selected for the relative comparison of the detected compounds.

For the lower limit of detection (LOD) and quantification (LOQ) estimation, we have used two compounds, 5-FU and FdUMP; where commercial standards are available, LOD was defined by 3×SD (standard deviation) and the LOQ by 10×SD [48,49]. The standard compounds were serially diluted in water as well as in processed conditioned media before analysis. The precursor m/z in the MS1 spectrum and fragment ion m/z were used for the identification of the compound from the lysate and media samples. Standards prepared in water are always clear and free from chemical noise, interfering contaminants that causes signal distortion on the MS. However, the challenges to detect and quantify the metabolites increases when trying to detect them in the complex biological matrices that includes numerous compounds with molecular weight similar or close to the target compounds thus creating difficulty in accurate identification or detection [50,51]. Hence, for the calibration curve, the standards were serially diluted (100 ng/µL to 0.195 ng/µL) in processed conditioned media samples before analysis. Using the stringent criteria, precursor and the fragment ions were detected, and the peak area was obtained using the Skyline v19.1 software. The calibration curve was generated for 5-FU and FdUMP, depicting the peak area of concentration from lowest (0.195 ng/µL also visually detected as LOD for 5-FU) to highest (25 ng/µL) (Figure 2). Quantification of the compounds were performed relatively to the peak area of FdUMP standard concentration of 0.780 ng/µL.

### 3.3. Method Development for the Identification and Quantification of 5-FU and Its Associated Metabolites

**Figure 3:**
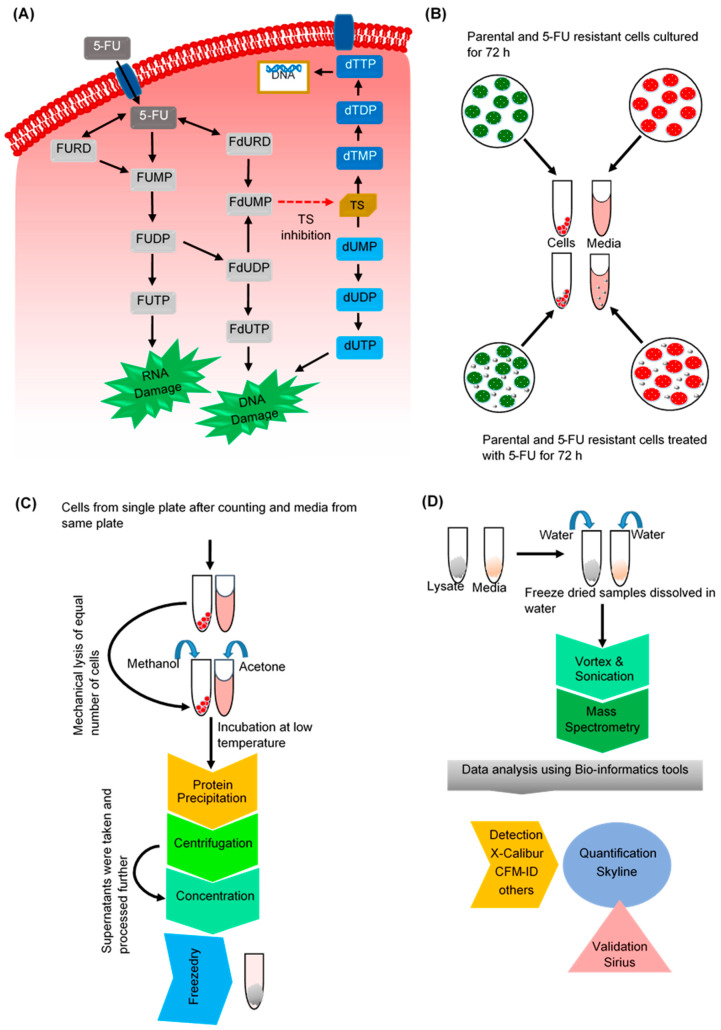
Development of quantitative metabolomics workflow to analyze 5-FU metabolism in CRC cells. (**A**) 5-Fluorouracil (5-FU) is an uracil analogue and within the cells it is rapidly converted into different metabolites. The active metabolites of 5-FU directly or indirectly affect replication and transcription leading the cells to undergo apoptosis. Following entry into the cells, 5-FU gets converted into fluorouridine (FURD), fluorouridine monophosphtate (FUMP) and fluorodeoxyuridine (FdURD). FURD gets converted into FUMP then to fluorouridine diphosphate (FUDP), which either subsequently forms fluorouridine triphosphate (FUTP) or gets converted into fluorodeoxyuridine diphosphate (FdUDP) and gets converted into FdUTP. FdUDP and the FdUDR gets converted into fluorodeoxyuridine monophosphate (FdUMP). FdUMP is a thymidylate synthase (TS) inhibitor that inhibits the synthesis of deoxythymidine triphosphate (dTTP) from deoxyuridine monophosphate (dUMP); hence, dUMP, in turn, can get converted into deoxyuridine triphosphate (dUTP). The FUTP, FdUTP and dUTP so formed gets incorporated into newly synthesizing DNA and RNA, leading to DNA and RNA damage. (**B**) Determination of changes in 5-FU and its metabolites in parental and 5-FU resistant LIM1215 cells was performed by quantitative metabolomic analysis. Parental and resistant cells were cultured until 70% confluency was reached and were treated with 50 µm 5-FU for 72 h. A comparative analysis needed the development of a protocol to effectively and efficiently extract 5-FU and its metabolites from within the cells as well as from the conditioned media with strategy for normalization of the results. The protocol shown was used to culture and treat the cells with equal volume of media with equal concentration of 5-FU. Cells were trypsinized, counted and extraction of compounds was performed following which the samples were freeze dried (**C**). (**D**) MS was coupled with F5 phase ultra high performance liquid chromatography (UHPLC) and data analysis was done using bioinformatics tools.

There have been several LC-MS based publications on the detection of 5-FU in cell culture [23,52]. These are basically low-resolution triple quadrupole MS-based, whereby the methods relied on available standards. The need for standards and low accuracy instrument therefore limits their use to screen for compounds where standards are not readily available. To overcome these limitations, we used high-resolution MS, which has been widely used in metabolomics [40] where resolution can be specified up to 240,000 with mass accuracies routinely <2 ppm without the need for external calibration standards. Within the cells, 5-FU gets converted into different metabolites (Figure 3A) and inhibition in the conversion of 5-FU into successive metabolites could be one of the reasons of chemoresistance. For the analysis of 5-FU metabolism, exploration of the status or levels of 5-FU and its metabolites in parental and 5-FU resistant cells were necessary. We developed an effective technique to extract and quantify 5-FU and its metabolites from the media and the cells to study the metabolism of 5-FU. This approach made use of existing publicly available information and bioinformatics tools to overcome some of the limitations associated with unavailability of standards. Effective extraction and quantification methods were developed, and for a comparative study, normalization was based on the equal cell number and equal volume of the culture media among the different groups of cells (Figure 3B–D).

The detection technique used was F5 phase UHPLC coupled to high-resolution tandem MS. With F5 phases, a total of four interaction mechanisms (polar, hydrophobic, aromatic and shape selectivity) are achievable to enhance separation of the polar 5-FU and its metabolites. The basic principle of this experiment involved separating the polar 5-FU and the metabolites in the first dimension before analyzing on the MS via ESI. Rather than analyzing all masses in a standard data dependent fashion (potentially missing out the compounds of interests), we created a preferred inclusion list and used the tSIM-ddMS2 methodology. Applying the MS in tSIM mode with a reduced mass range in the quadrupole allows us to increase the signal to noise ratio of the analytes, and thus, better detection limits. Once the analyte of interest is observed through the accurate mass measurement, an MSMS event is triggered to obtain the fragment ion. Therefore, for accurate identification it was essential to set up a fingerprint for each compound based on the both the accurate precursor mass and fragment ions produced by dissociation of precursors.

**Figure 4:**
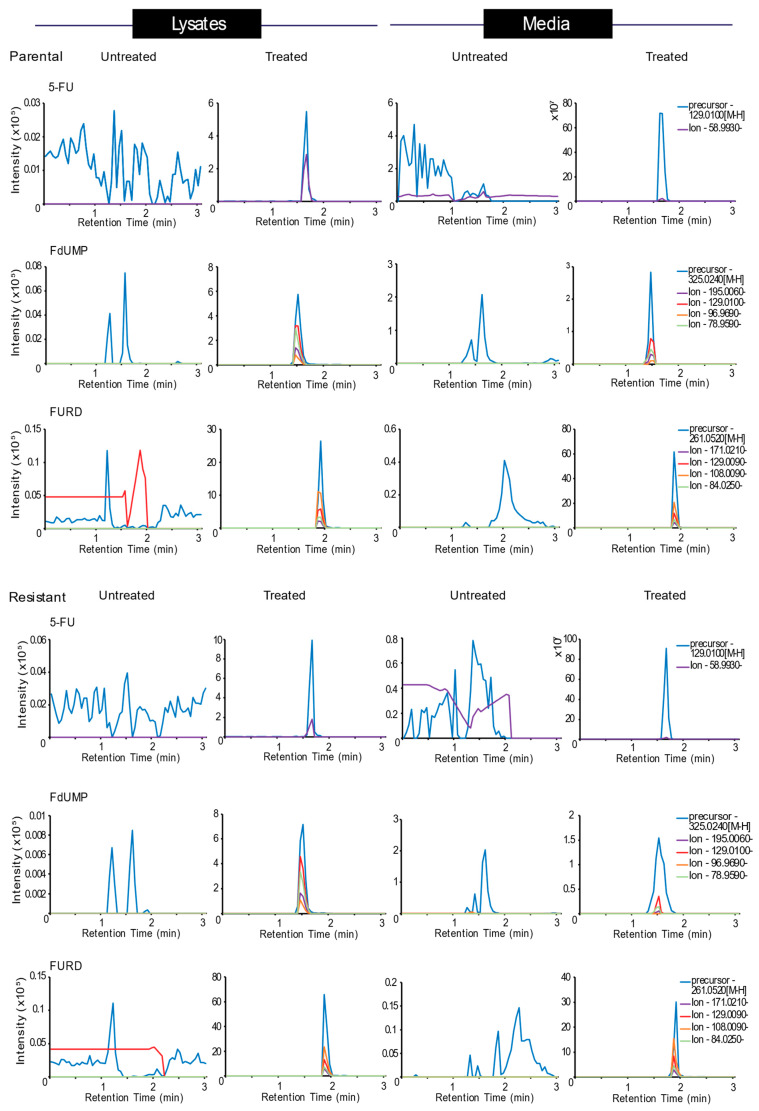
Relative quantitation of 5-FU and its metabolites.

Fragment ions were selected by analyzing the experimentally generated data from standard samples (5FU and FdUMP), theoretically predicted m/z spectrum using online databases for compounds whose standards were not available (FURD, FdURD, FUTP, FdUTP, dUMP and TMP) and predicted precursor m/z based on machine learning algorithms. The selected precursor m/z and target compounds m/z with the gas phase structure for detected compounds (5-FU, FdUMP, FURD, FdURD, dUMP and TMP) of these are shown in Supplementary Figure S3. In the Skyline v19.1 software, the molecular fingerprints were used to view and quantify individual compounds based on the precursor and fragment peaks and the presence of the peaks only in the treated samples. The representative extracted chromatograms for the detected and quantified compounds whose standards were available (5-FU and FdUMP) as well as whose standards were not available (FURD) comparative to untreated samples between parental and resistant cell lysates and media samples are shown in Figure 4. The chromatograms for other FdURD, dUMP and TMP compounds are shown in Supplementary Figure S4.

To detect and quantify the target compounds in the media and lysates, Skyline software was used. In each individual sample, the precursor and fragment ion peaks were searched for. Treated samples showed the extracted ion chromatogram peaks specific for precursors and the fragment ions from MS2. The fragmentation ion generated were aligned along the same retention time as the precursor. Elution from F5 phase UHPLC at lower retention time, signifying that the compounds are polar in nature. Absence of the peaks in the untreated sample validated the accurate detection of the compound. The figure shows chromatograms for 5-FU, FdUMP and FdURD identified in treated samples, which were not observed in untreated samples. The precursor peaks are colored in blue and fragment ions are presented with varying colors. The chromatograms are representative of 10 biological replicates.

### 3.4. Further Validation of the Accurate Identification of the Compounds

Observation of extracted ion chromatogram peaks representing the molecular fingerprints for each compound and detection only in the treated sample were evidences of positive identification. However, to further validate that the detection and identification of the compounds were accurate, reproducible and reliable and not any other compounds of similar molecular weight, we have utilized the Sirius software where both isotope pattern analysis and information from fragmentation were used for calculation and identification. The precursor and product mass spectrum data were compared independently to the PubChem database and all molecular formula settings within the software to view the identification score for each compound. The scores represent the probability and higher score represent the chance that molecular formula is correct while the tree score is the probability that the molecular formula would generate the predicted data.

**Figure 5:**
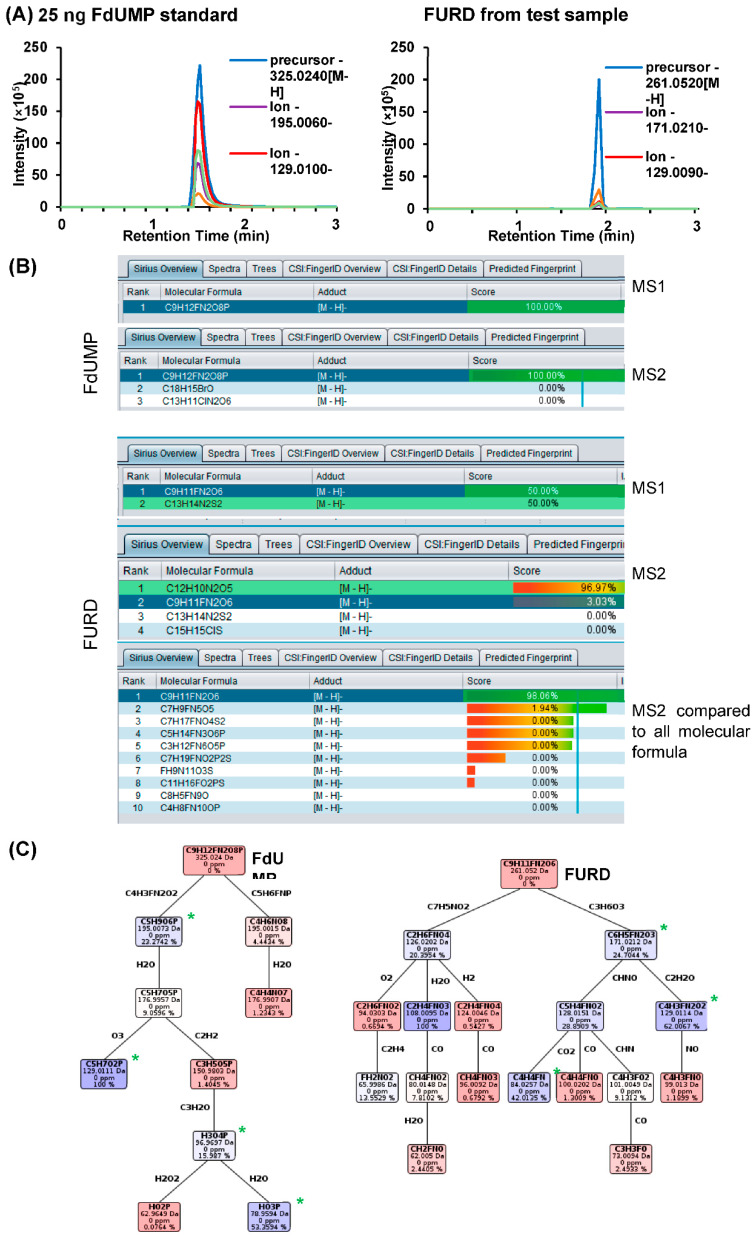
Isotope pattern analysis and fragmentation tree analysis using SIRIUS. To further validate the results, the experimental prediction was compared with theoretical prediction and database searches using Sirius software. The m/z for both precursor and the product ions were subjected to analysis using SIRIUS, which compared the data to the PubChem as well as all the molecular formula of similar masses to provide an identification score. Example shown here was using FdUMP standard and FURD, whose standard was not available. The chromatograms depicting precursors and fragment ions peaks for FdUMP standard and FURD from test sample (**A**). (**B**) Result obtained from SIRIUS software following the comparison of the MS1 and MS2 spectrum derived from Skyline with PubChem only for FdUMP and PubChem and all possible molecular formula for FURD (MS2). Both comparisons from MS1 and MS2 gave positive score predicting correct identification for all compounds and all the results were within the acceptable mass deviation of 5 ppm. (**C**) The tool also predicts the fragment ions that could have been generated from the predicted molecular formula and represented as a fragmentation tree that explains the molecular formula for the selected fragment ions. ‘*’ represents the ion fragments that were used for the identification of the compounds that were successfully predicted with SIRIUS 4.0.1 as the fragment ion generated from dissociation of the precursor compound.

As shown in Figure 5, the data in the form of chromatograms could be obtained from skyline software for FdUMP standard and FURD, whose standard was not available. The chromatograms are intensity peaks for precursor and fragment ions generated from the specific precursors (Figure 5A). The spectral data derived from skyline was imported into SIRIUS 4.0.1 software and the identification was attempted for all compounds. Representative results obtained for FdUMP and FURD as identification scores and fragmentation tree predicted for these are shown in the Figure 5B,C. Table 4 shows the positive identification score for the compounds identified with the data compared to both PubChem database and all molecular formula. The mass deviation was within the acceptable range of 5 ppm. The fragmentation tree (Figure 5C) is the representation of the prediction of the fragment ions that might generate from the given precursor and the results show that the transition masses that were selected for the identification of the compounds were accurately predicted by SIRIUS 4.0.1 software.

### 3.5. 5-FU Metabolism is Impaired in Resistant CRC Cells

**Figure 6:**
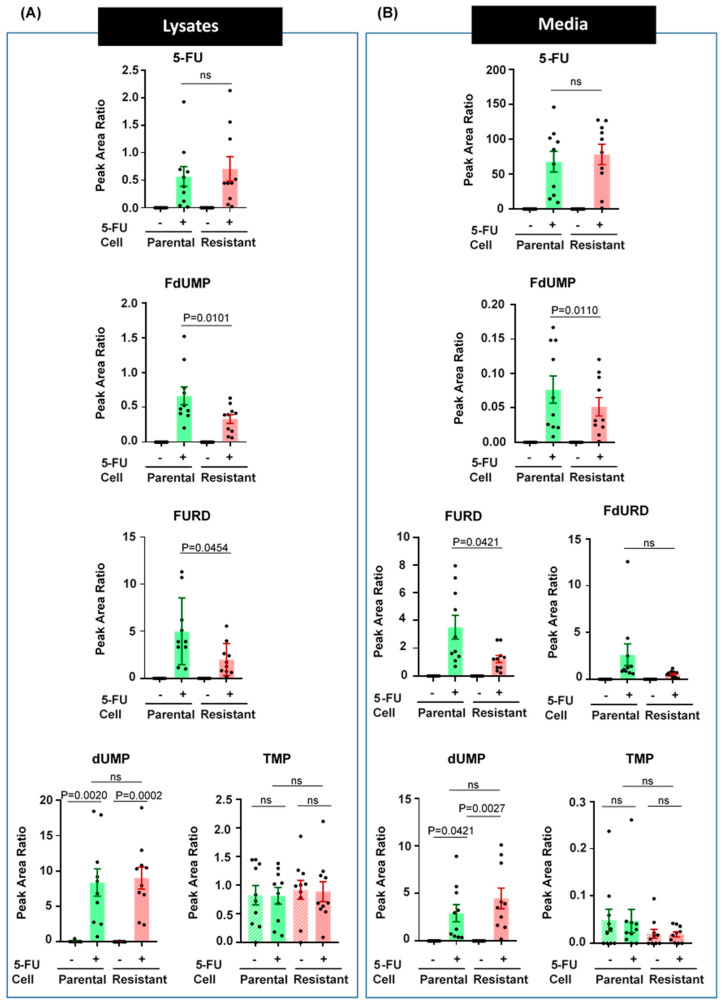
5-FU metabolism is dysregulated in resistant cells. To analyze the metabolism pathway of 5-FU in parental and resistant cells, LIM1215 cells were cultured in media with and without 50 µM 5-FU for 72 h. Media and cells were collected, and the metabolites were extracted. Trypan blue cell counting was used to determine the cell concentration and an equal number of cells were resuspended in methanol and mechanically lysed to extract the metabolites. Samples were subjected to tandem mass spectrometry coupled to F5 phased UHPLC. Raw data were processed and analyzed using bioinformatics tools, particularly, Skyline. Quantitation was based on the peak area obtained from Skyline and the comparison was normalized relative to the peak area of 0.780 ng/µL FdUMP standard. The graphs represent levels based on the peak area ratio of 5-FU and reduced levels of metabolites in resistant cells compared to parental cell lysates (**A**) and media (**B**), revealing that the 5-FU metabolism pathway is impaired in resistant cells. ns; not significant. Data is presented as mean ± s.e.m. determined by Student’s t-test (n = 10).

For understanding the mechanism of resistance, it was necessary to analyze the metabolism pathway of 5-FU. To ensure accurate measurements, the analysis was performed multiple times with 10 biological replicates. The data were consistent for each replicate with minor differences among the measurements signifying the reproducibility and efficiency of the methodology. The levels of 5-FU and its metabolites including levels of the endogenous nucleotides related to the metabolism pathway were determined. The quantitative metabolomics approach targeted 5-FU and its metabolites FdUMP, FURD and FdURD as well as two endogenous nucleotides, dUMP and dTMP. Levels of 5-FU were not significantly different in resistant cell lysate and media compared to samples from parental cell lysate and media suggesting there was no difference in the level of 5-FU inside the cells. However, the levels of metabolites FdUMP and FURD were significantly lower in the 5-FU resistant cells compared to parental cell lysates (Figure 6A) and media (Figure 6B) samples. FdURD was not detected in cell lysate but was present abundantly in media samples and the level was lower in media from resistant cells compared to parental conditioned media. Levels of dUMP was elevated, although not significant in media from resistant cells. Overall, both nucleotides, dUMP and TMP, were present in lysates and media with no significant difference in levels among the lysates. On the contrary, levels of TMP were not changed following treatment with 5-FU. Taken together, these results suggest that CRC 5-FU resistant cells have a defective metabolism especially in the conversion of FdUMP and FURD.

### 3.6. FdUMP Sensitises 5-FU Resistant CRC Cells

**Figure 7:**
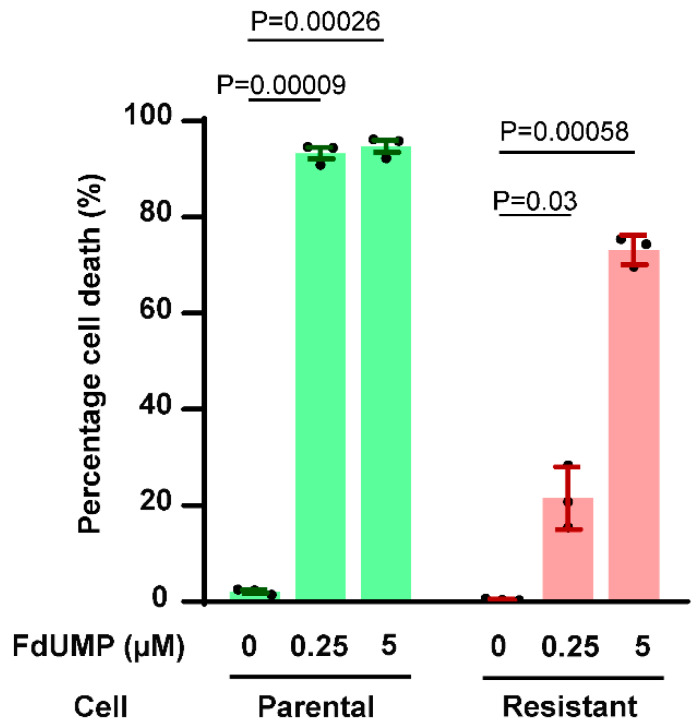
Resistant cells are sensitive to active metabolite FdUMP.

Elucidation of the impaired metabolic pathway clarifies that the active metabolites are not formed in the resistant cells, which prevents the cells from 5-FU mediated cell death. In this scenario, if the cells are subjected to treatment with the active metabolites directly, they should be sensitive to the treatment validating that the metabolism pathway is indeed obstructed in resistant cells. To determine whether supplementing the active metabolite on its own, bypassing the complete 5-FU metabolism pathway, could induce cell death, parental and 5-FU resistant cells were treated with a low (0.25 µM) and high (5 µM) concentration of FdUMP for 72 h. The percentage of cell death was measured using FACS apoptosis assay. While 5-FU did not sensitize LIM1215 5-FU-resistant CRC cells (Figure 1B), FdUMP at both low and high concentration was able to sensitize the cells and induce cell death in vitro (Figure 7). Importantly, even low concentrations of FdUMP was able to significantly sensitize the 5-FU resistant cells thereby highlighting the defect in 5-FU metabolism in chemoresistant cells (Figure 7).

Parental and 5-FU resistant cells were treated with relatively low 0.25 nm and high 5 nm concentrations of fluorodeoxyuridine monophosphate (FdUMP). Graph represents the percentage cell death analyzed using propidium iodide uptake followed by FACS. Results show FdUMP induces cell death significantly in parental and resistant LIM1215 cells. Parental cells showed high sensitivity to FdUMP, while resistant cells appeared more sensitive to higher concentration. Data is presented as mean ± s.e.m. determined by Student’s t-test (n = 3).

## 4. Discussion

Extraction of 5-FU metabolites and their quantification from cultures and human samples has been previously described by different studies [23,37]. In this study, we developed a protocol in the absence of standards to suit all adherent cell types and at the same time would be effective for the quantitative comparison of the metabolites among multiple groups (parental and 5-FU resistant CRC cells in presence and absence of 5-FU). Even though the study has limitations, such as using only one cell line for the protocol development, we attempted to further improve the separation and detection of the compounds by using UHPLC coupled to MS. Given that isotopically labelled standard samples are not available for all involved targets, it was essential to establish a method for comparative study. Relative quantification thus has to utilize multiple approaches such as instrument’s high mass accuracy and resolution, targeted screening methodology, prior information in literature and bioinformatics [53].

5-FU and its metabolites are hydrophilic and the endogenous nucleotides, dUMP and TMP, are polar because of the phosphate group; hence, reversed phased liquid chromatography would be posed with a challenge for their separation [23], whereas hydrophilic interaction chromatography suffers from matrix effects and peak shape distortion when loading samples at high acetonitrile concentrations. We therefore approached the separation by using F5 phase, which is ideal for polar nucleotide and metabolites separation [24,54]. For the quantitative MS, various methods have been described, such as multiple reaction monitoring (MRM) [55] and selected reaction monitoring (SRM), single reaction monitoring (SIM), and in this study, a high-resolution based targeted, t-SIM ddMS2 setup was used to provide enhanced selectivity and sensitivity [21,22].

The detection and identification of the analytes are preliminary challenges that any experiment needs to overcome before quantification. Here, we discussed two methods that could be implemented in two different cases for the identification of the compounds. The first one is the use of available standards to generate molecular fingerprints, such as the precursor m/z, their fragment ions m/z and the characteristic retention time. 5-FU and FdUMP in culture media and cell lysate were readily detected and identified based on this available information. Second, for the cases where the standard samples were not available, the identification and detection can be based on the detection of the precursor isotopes in the MS1 scan using the measured accurate masses plus the accurate detection of the daughter ion fragments in the MS2 spectrum matching the theoretically predicted m/z fragments as obtained from CFM-ID, HMDB [32,35]. The cell lysate and media samples consist of mixture of unrelated compounds of similar m/z; hence, for accuracy, the negative samples were ran together and the fragment ion peaks that were common in both treated and untreated samples were ruled out and only the peaks present in the treated samples were considered, as performed for other metabolites in this study.

The concentrations of 5-FU metabolites were observed to be varying among the two groups of cells. Importantly, the levels of 5-FU metabolites FdUMP and FURD were found to be lower in resistant cells compared to parental. Studies have shown that the level of FUTP is higher in the Peripheral Blood Mononuclear Cells (PBMCs), while the level of other metabolites are speculated to be low [56]. However, in this study, FUTP and FdUTP were not detected, which could be due to low levels below the detection limit. Further studies with the specific standard samples and/or similar extraction methodologies might be helpful for the detection of such metabolites.

All the results of the metabolomics clearly validated that 5-FU metabolism is in turn inhibited in the 5-FU resistant cells. The other possible mechanism includes the effective efflux of drugs and metabolites from within the cells via the transporter proteins such as ATP-binding cassette proteins (ABC proteins) [57] to extracellular matrix before the metabolites interfere with transcription and translation. The elevated level of FdUMP and FURD in media of resistant cells compared to parental shows that the metabolites could also be actively effluxed from the cells and hence contribute to resistance [58,59,60]. In this study, supplementing the cells with FdUMP sensitized the resistant cells, which clarified that the resistant cells may have impaired 5-FU metabolism or drug efflux. However, it is noteworthy that the treatment of cells with FdUMP showed some degree of difference in sensitivity among parental and resistant cells. The resistant cells were comparatively less sensitive than parental cells, which suggests that there might be additional factors contributing to the resistance of the cells other than 5-FU metabolism defect. Consistent with our results, a previous study showed that 10-mer of FdUMP is more potent than 5-FU in reducing cell proliferation in NCI 60 cell line screen and also showed effective action in vivo by reducing the tumor burden in CRC xenograft mouse models [61]. As this study was conducted for the first time in 5-FU resistant CRCs, it is evident that the resistance is indeed attributed by a defect in the metabolism of 5-FU in cancer cells. Hence, the results highlight the utility of FdUMP as a potential therapeutic agent for 5-FU resistant CRC patients.

## 5. Conclusions

Altogether, the multi-approach protocol developed in this study could be used for effective detection and quantification of 5-FU and its associated metabolites from cell cultures. This protocol using UHPLC coupled with high-resolution MS is a very sensitive, accurate and reliable approach for quantitative metabolomics. Importantly, the protocol discussed in this study can be used for quantitative metabolomics analysis in presence or absence of the standards for the target compounds. In addition, the protocol described can be used to examine the metabolism or pharmacological response of certain polar compounds in the cells. Slight modification in the protocol will hopefully be beneficial in the studies where the metabolites are to be quantified from suspension cells as well as the biofluids or other biological samples.

Furthermore, the study clarified that 5-FU resistant cells resist 5-FU by inhibiting the metabolism of 5-FU, thereby preventing the DNA and RNA damage, which could be effectively solved by treating the resistant cells with the active metabolite FdUMP. Indeed, further preclinical studies need to be carried out to test the in vivo efficacy of FdUMP in CRC patients who are not responding to 5-FU.

## Figures and Tables

**Table 1:** Parameters on the QE HF Orbitrap utilizing the tSIM-ddMS2 methodology.

**tSIM Settings**	
MS1 resolution	120,000
MS1 AGC target	200,000
Max IT time (ms)	200
Isolation window	4.0 m/z
Loop count	4
Isolation width	
**ddMS2 Setting**	
HCD NCE	25
Loop count	4
MS2 resolution	15,000
MS2 AGC target	200,000
Max IT time (ms)	45
Isolation window	1.2 m/z
Charge exclusion	>2

**Table 2:** List of target compounds. Precursor m/z calculated using isotope simulation and filter applied to visualize their fragments in Xcalibur Qual browser.

Target	Molecular Formula	Molecular Weight (g/mol)	m/z [M-H]-	Scan Filter
5-FU	C_4_H_3_FN_2_O_2_	130.078	129.0109	127.0090–131.0090
FdUMP	C_9_H_12_FN_2_O_8_P	326.173	325.0211	323.0240–327.0240
FURD	C_9_H_11_FN_2_O_6_	262.193	261.0529	259.0520–263.0520
FdURD	C_9_H_11_FN_2_O_5_	246.194	245.0605	243.0570–247.0570
FUTP	C_9_H_14_FN_2_O_15_P_3_	502.13	500.9517	498.9510–502.9510
FdUTP	C_9_H_14_FN_2_O_14_P_3_	486.131	484.956	482.9560–486.9560
dUMP	C_9_H_13_N_2_O_8_P	308.183	307.0338	305.0330–309.0330
TMP	C_10_H_15_N_2_O_8_P	322.21	321.0497	319.0480–323.0480

**Table 3:** The accurate mass of the precursor and fragment ions (fingerprint) used to identify 5-FU and the associated seven metabolites. The retention times that the targets were observed and detected in are shown in middle column in minutes, being polar in nature the compounds got eluted in lower retention time. ‘–‘ represents no detection.

Compound	Molecular Weight (g/mol)	Retention Time (min)	Precursor (m/z)	Fragment (m/z)
5-FU	130.0173	1.6	129.01	58.993
FdUMP	326.0313	1.4	325.024	195.006
				129.01
				96.969
				78.959
FURD	262.0593	1.9	261.052	171.021
				129.009
				108.009
				84.025
FdURD	246.0643	2.1	245.057	155.025
				129.009
				112.0204
FUTP	501.9583	-	500.951	482.9407
				158.900
				129.01
FdUTP	485.9633	-	484.956	441.9506
				256.80
				129.01
dUMP	308.0403	1.4	307.033	195.006
				111.02
				96.9696
TMP	322.0553	1.5	321.048	195.005
				125.0355
				96.9691

**Table 4:** SIRIUS scores validating the correct identification of the compounds. To validate that the compounds were identified correctly, MS1 and MS2 data exported from the skyline chromatograms were analyzed using the Sirius software. The data were compared with the PubChem database as well as with all the molecular formula options within the software and scores were obtained. Detection showed positive score for all detected compounds with the mass deviation within the acceptable range of ‒5 to 5 ppm. The tree score represents the positive fragment ions predicted for each compound.

Sirius Compound Prediction						
Compound	PubChem			All Molecular Formula		
	Score	Tree Score	Median mass Deviation (ppm)	Score	Tree Score	Median Mass Deviation (ppm)
Standards (25 ng)						
FdUMP Precursors	100		−3.75	12.05		−3.57
FdUMP Products	100	37.55	0.27	14.41	14.75	0.78
5-FU Precursor	33.33		0.43	97.91	0	0.43
5-FU Products	99.97	8.06	−2.21	100	15.45	3.07
Test Samples						
5-FU Precursor	33.33		0.75	97.91		0.75
5-FU Products	99.84	7.22	−4.6	100	27.22	−2.35
FdUMP Precursors	100		−3.57	12.05		−3.57
FdUMP Products	100	27.34	−2.2	0.46	18.83	−2.97
FURD Precursor	50		−0.65	43.67		−0.65
FURD Products	3.03	106.22	−2.96	98.06	53.99	1.81
FdURd Precursor	12.8		−0.2	77.78		−0.2
FdURD Products	86.8	90.1	−3.15	97.68	72.41	3.3
dUMP Precursor	20		0.8	12		0.8
dUMP Products	99.3	52.65	1.1	100	41.99	−0.5
TMP Precursors	12.5		1.51	7.96		1.28
TMP Products	100	31.59	0.69	62.18	31.59	0.69

## Supplementary Materials

The following are available online at https://www.mdpi.com/2079-7737/9/5/96/s1. Figure S1: FURD detection using X-caliber Qual browser, Figure S2: FdUMP detection using X-caliber Qual browser, Figure S3: Molecular structures, Figure S4: Chromatograms depicting identification of FdURD, dUMP and TMP from cell lysates and media samples from parental and resistant cells treated with 5-FU, Figure S5: Isotope pattern analysis and fragmentation tree analysis for 5-FU standard using SIRIUS.
