# Supplementary material for: A High-Resolution Mass Spectrometry-Based Quantitative Metabolomic Workflow Highlights Defects in 5-Fluorouracil Metabolism in Cancer Cells with Acquired Chemoresistance
Source: Biology (Basel). 2020 May 6;9(5):96. doi: 10.3390/biology9050096 (PMC7284906; doi:10.3390/biology9050096)
Supplement: Supplementary file 1 [file biology-09-00096-s001.pdf]

Supplementary Figure 1

A

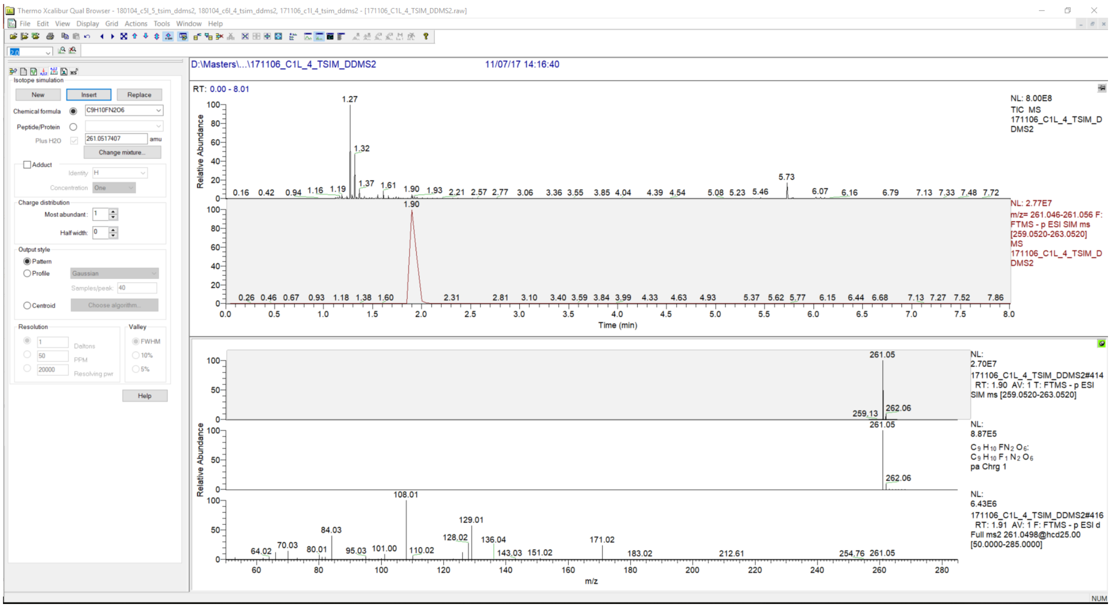

B

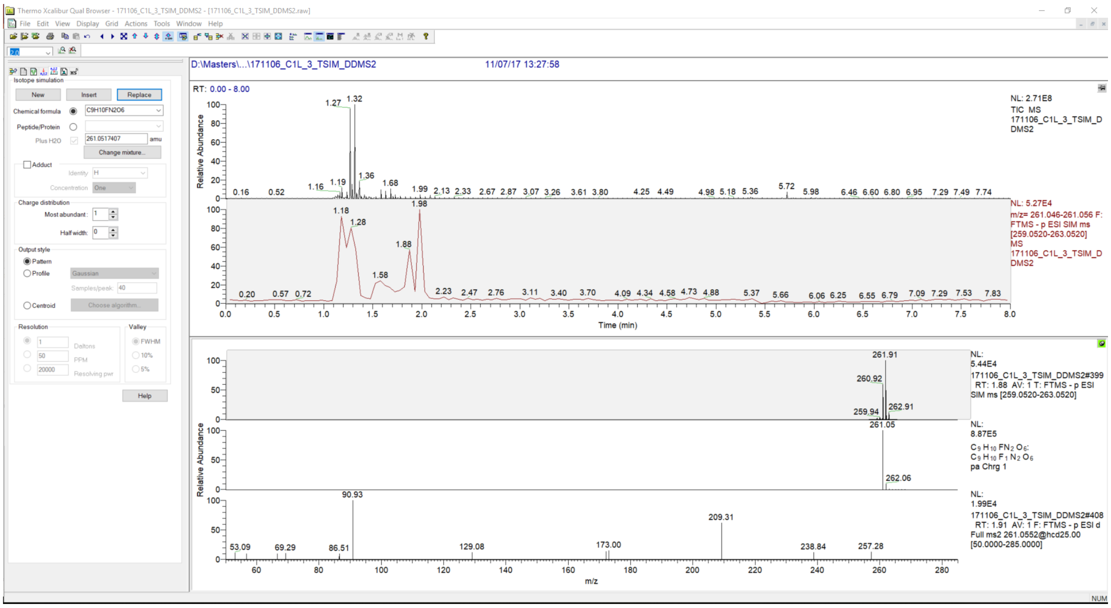

Supplementary Figure. 1. FURD detection using X-caliber Qual browser

(A) The precursor isotopes m/z for FURD calculated using the molecular formula in isotope simulation tool of X-caliber Qual browser on the left pane of the screenshot. The top spectrum is the spectrum obtained from the complete MS. The lower spectrum on the top half of the picture is the peaks that match the target m/z. The lower top spectrum represents the ions of similar size that could be potential isotopes. The middle spectrum represents the theoretically calculated precursor isotope m/z values (removal of one H-atom from the molecular formula to represent the negative ion mode or deprotonation) and the bottom spectrum represents the MS2 of specific target precursor m/z. (B) Data analyzed from the untreated sample in the exact same way showing mismatch of the precursor and the fragment ions.

A

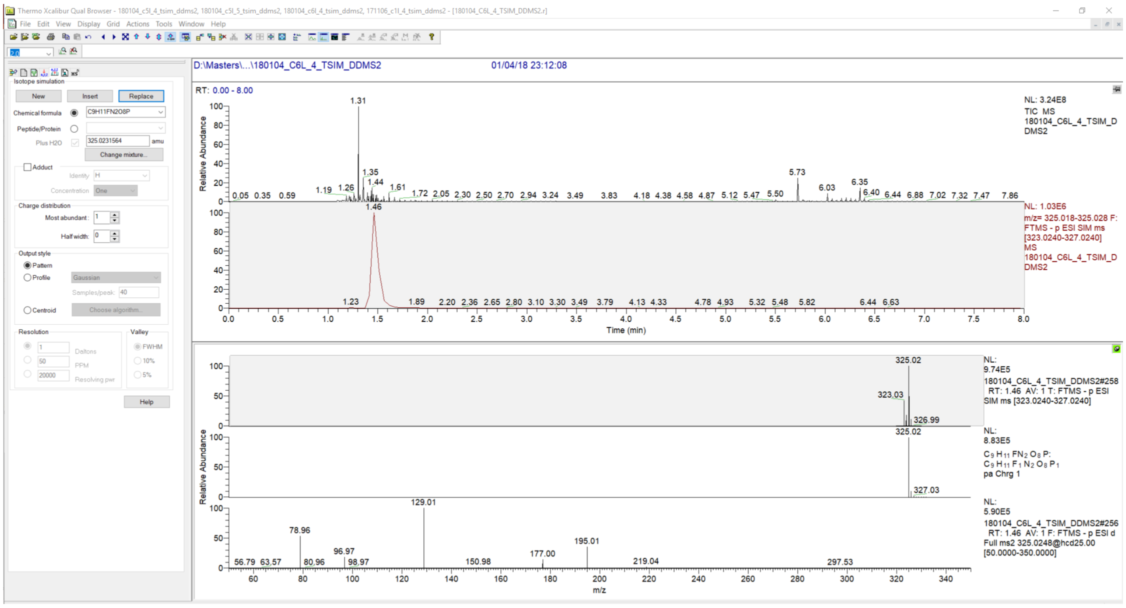

B

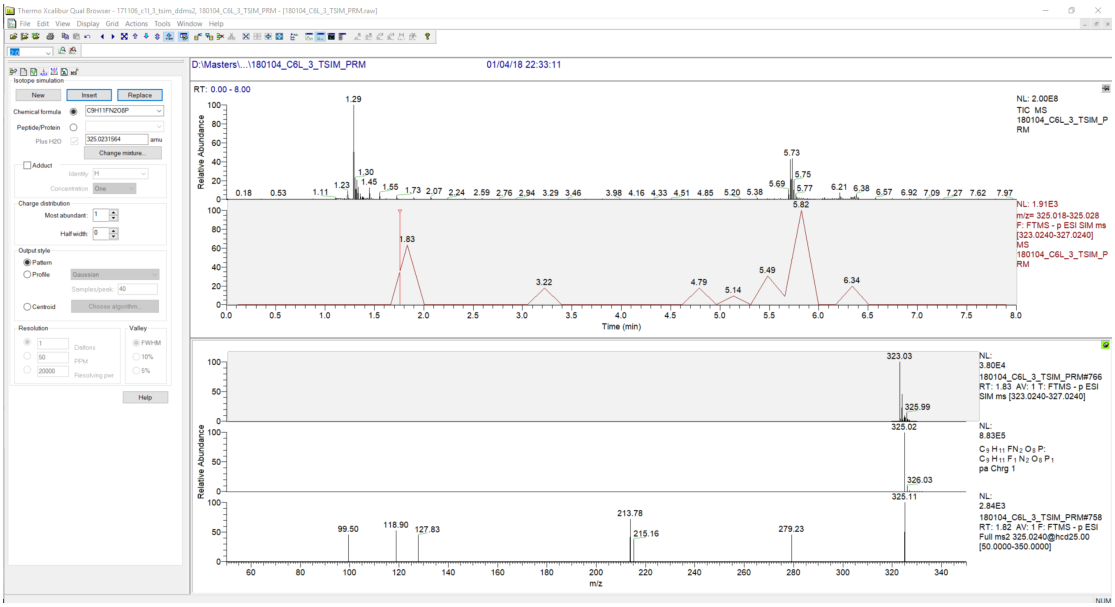

Supplementary Figure. 2. FdUMP detection using X-caliber Qual browser
(A) The precursor isotopes m/z for FdUMP calculated using the molecular formula in isotope simulation tool of X-caliber Qual browser on the left pane of the screenshot. The top spectrum is the spectrum obtained from the complete MS. The lower spectrum on the top half of the picture is the peaks that match the target m/z. The lower top spectrum represents the ions of similar size that could be potential isotopes. The middle spectrum represents the theoretically calculated precursor isotope m/z values (removal of one H-atom from the molecular formula to represent the negative ion mode or deprotonation) and the bottom spectrum represents the MS2 of specific target precursor m/z. (B) Data analysed from the untreated sample in the exact same way showing mismatch of the precursor and the fragment ions.

5-FU

Canonical SMILES : C1=C(C(=O)NC(=O)N1)F

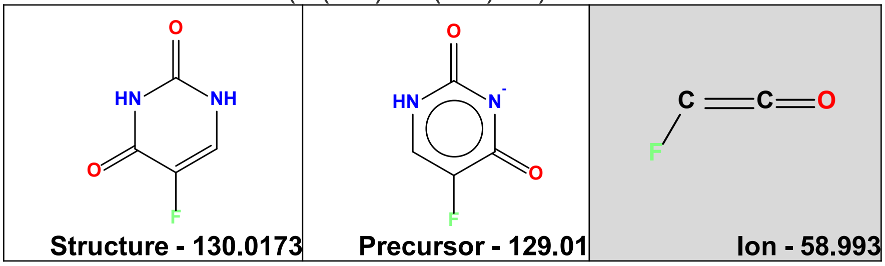

FdUMP

Canonical SMILES : C1C(C(OC1N2C=C(C(=O)NC2=O)F)COP(=O)(O)O)O

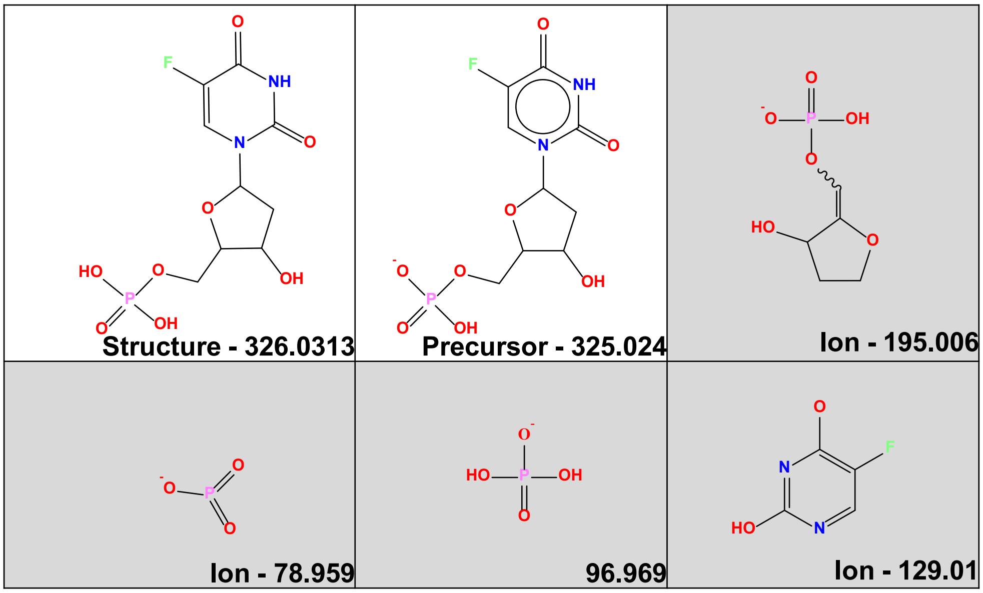

FURD

Canonical SMILESz : C1=C(C(=O)NC(=O)N1C2C(C(C(O2)CO)O)O)F

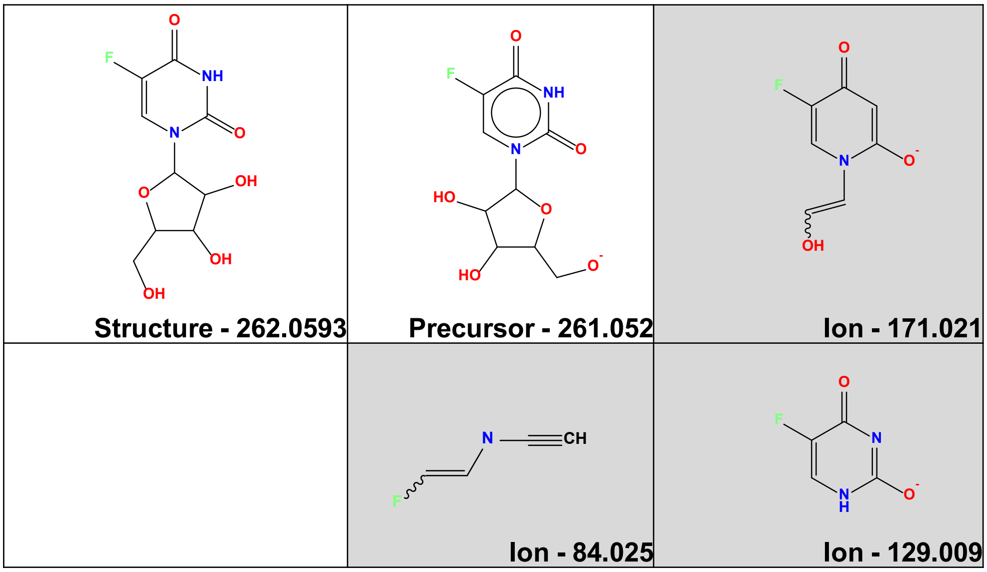

Supplementary Figure 3

FdURD
Canonical SMILES: C1C(C(OC1N2C=C(C(=O)NC2=O)F)CO)O

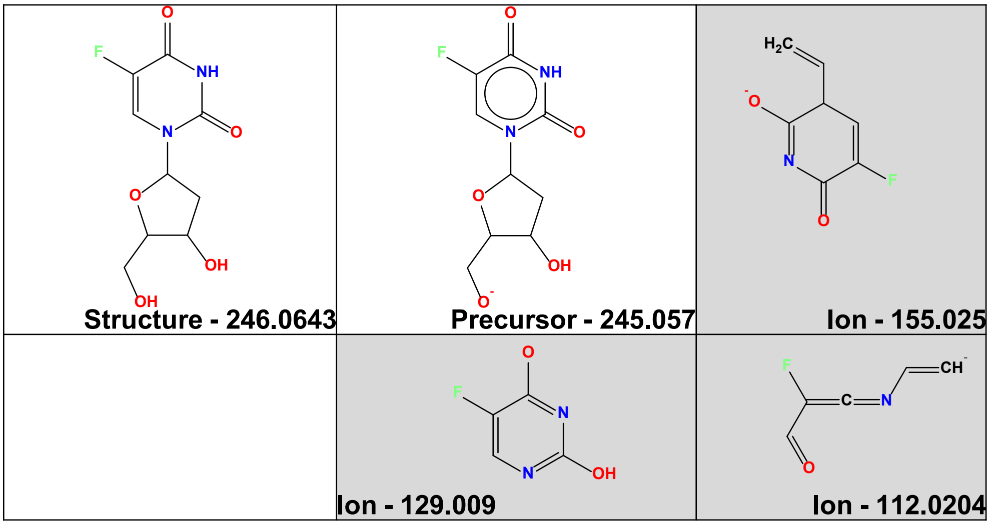

TMP
Canonical SMILES: CC1=CN(C(=O)NC1=O)C2CC(C(O2)COP(=O)(O)O)O

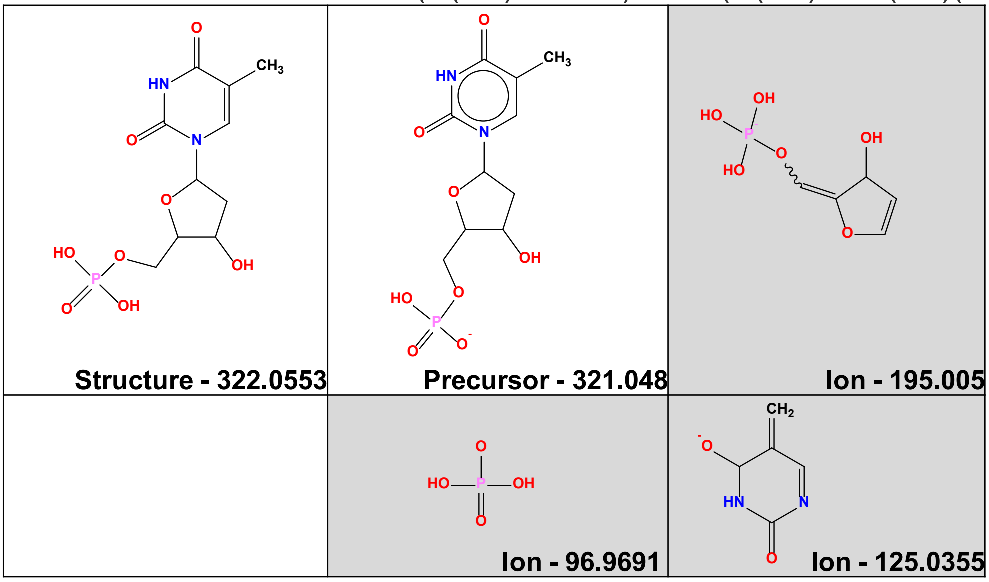

dUMP
Canonical SMILES: C1C(C(OC1N2C=CC(=O)NC2=O)COP(=O)(O)O)O

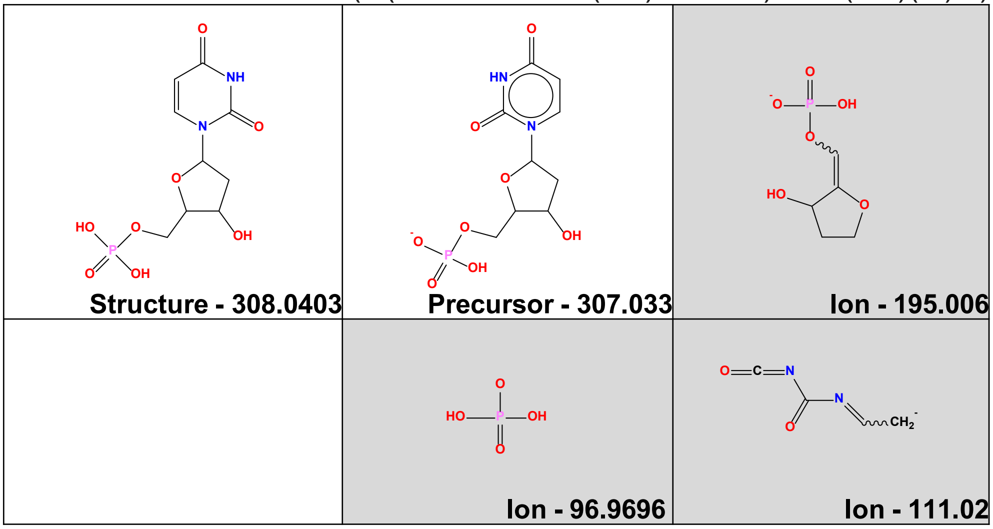

Supplementary Figure. 3. Molecular structures

Molecular structures and accurate mass of both the precursor and fragmentation masses of the 8 compounds. The predicted fragmentation ion and mass generated by the CFM-ID and was cross checked with HMDB. The structures are shown only for the experimentally detected ions which were explained by bioinformatics tools CFM-ID and HMDB.

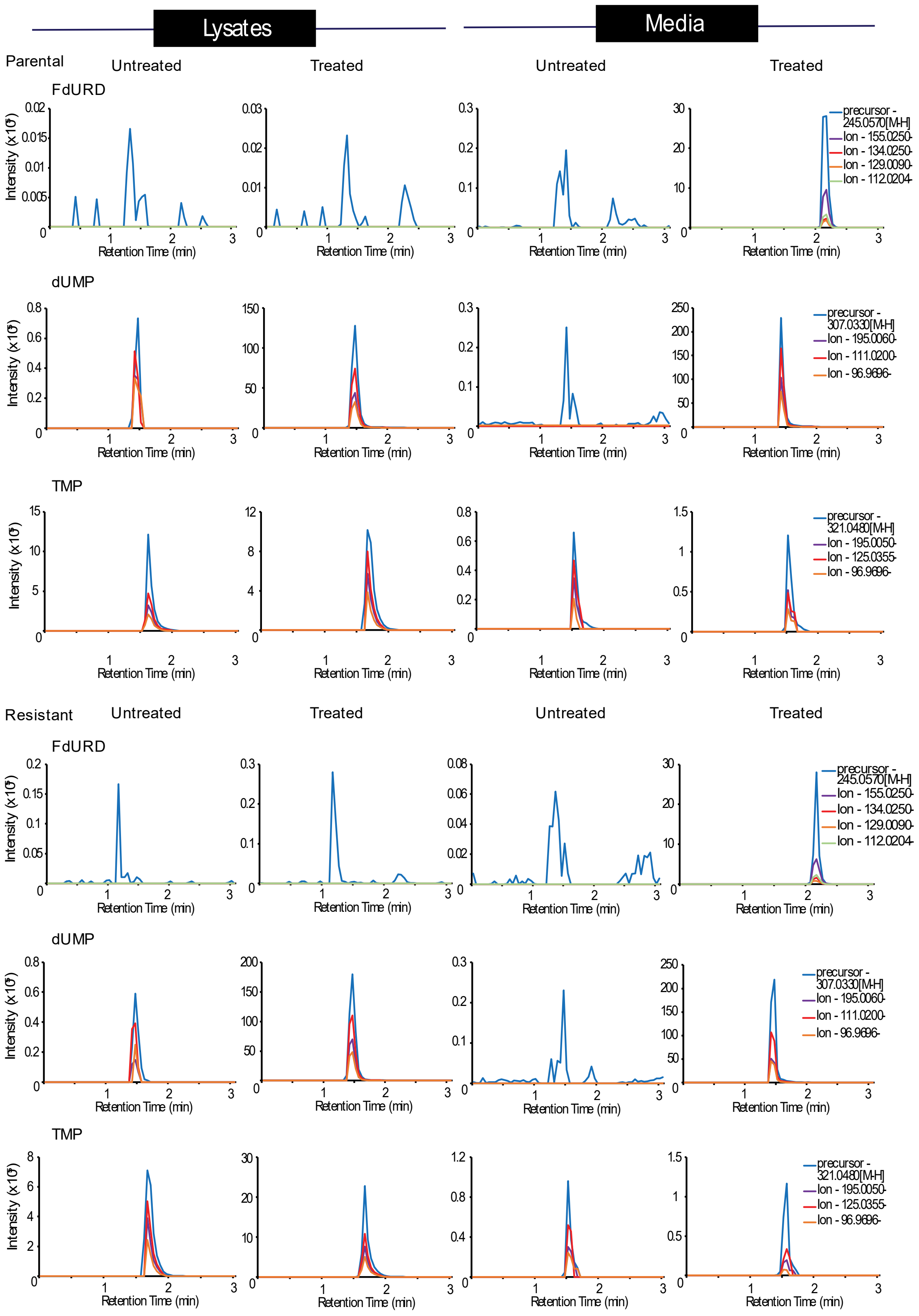

Supplementary Figure. 4. Chromatograms depicting identification of FdURD, dUMP and TMP from cell lysates and media samples from parental and resistant cells treated with 5-FU.

Supplementary Figure 5

5- FU

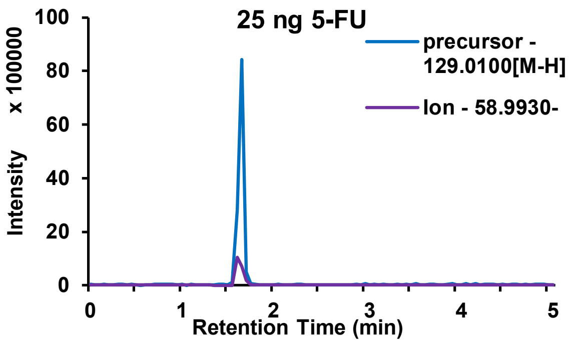

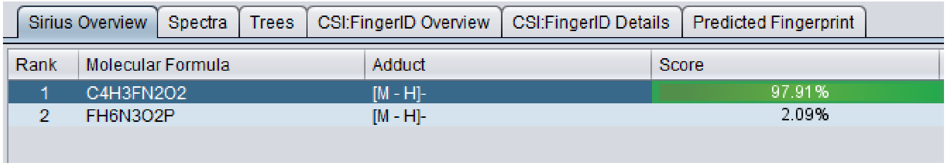

Sirius Overview | Spectra | Trees | CSI:FingerID Overview | CSI:FingerID Details | Predicted Fingerprint

Rank	Molecular Formula	Adduct	Score
1	C4H3FN2O2	[M - H]-	97.91%
2	FH6N3O2P	[M - H]-	2.09%

MS1

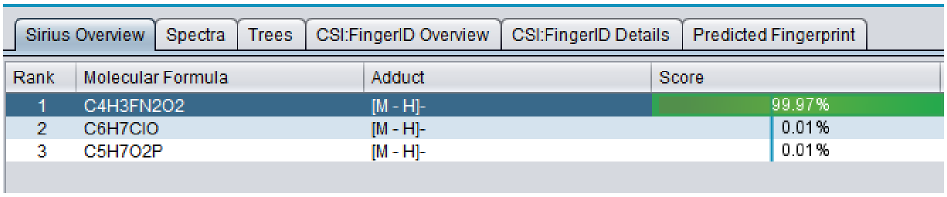

Sirius Overview | Spectra | Trees | CSI:FingerID Overview | CSI:FingerID Details | Predicted Fingerprint

Rank	Molecular Formula	Adduct	Score
1	C4H3FN2O2	[M - H]-	99.97%
2	C6H7ClO	[M - H]-	0.01%
3	C5H7O2P	[M - H]-	0.01%

MS2

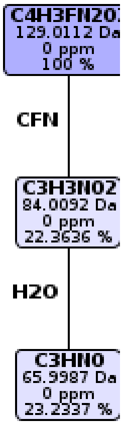

Supplementary Figure. 5. Isotope pattern analysis and fragmentation tree analysis for 5-FU standard using SIRIUS.
